# A Challenging Diagnosis of Steroid-Responsive Encephalopathy Associated With Autoimmune Thyroiditis (SREAT) in a Systemic Lupus Erythematosus (SLE) Patient With Hashimoto Encephalopathy (HE): A Case Report

**DOI:** 10.7759/cureus.73262

**Published:** 2024-11-07

**Authors:** Radwa Genidy, Aymen Abbas, Abd Al Kareem Adi, Shazia Abdullah, Ahmad Nizam

**Affiliations:** 1 Internal Medicine, Sheikh Khalifa Medical City, Abu Dhabi, ARE; 2 Rheumatology, Sheikh Khalifa Medical City, Abu Dhabi, ARE; 3 Neurology, Sheikh Khalifa Medical City, Abu Dhabi, ARE

**Keywords:** anti-double-stranded dna, hashimoto encephalopathy, hashimoto thyroiditis, neuropsychiatric systemic lupus erythematosus (npsle), sreat, systemic lupus erythematosis

## Abstract

Steroid-responsive encephalopathy associated with autoimmune thyroiditis (SREAT) is a rare condition that can present with multiple neurological and psychiatric manifestations. SREAT diagnosis poses a challenge due to the nature of its nonspecific symptomatology and its overlap with numerous autoimmune, metabolic, infectious, and neuropsychiatric disorders. It is associated with elevated anti-thyroid antibodies, occurs in correspondence with autoimmune thyroiditis, and shows great response to corticosteroid treatment. We present the case of a 27-year-old female patient with a complex medical history including systemic lupus erythematosus (SLE) and autoimmune thyroiditis. She presented to the hospital with bizarre behavior, psychosis, and confusion. Urine toxicology screen and septic workup were negative. Neuropsychiatric SLE (NPSLE) was high on the list of differential diagnosis but was excluded as the patient's previous lupus flares demonstrated an upward trend in anti-double-stranded deoxyribonucleic acid antibody (anti-dsDNA Ab) titers and low complement levels and leukocyte counts which were not present during this admission. Her cerebrospinal fluid (CSF) analysis was unremarkable except for higher-than-normal immunoglobulin G4 (IgG4) levels. Brain magnetic resonance imaging (MRI) was normal, and magnetic resonance angiography (MRA)/magnetic resonance venography (MRV) studies were unremarkable. Serum anti-thyroid antibodies were elevated which led to the consideration of Hashimoto encephalopathy (HE). SREAT diagnosis was made, and she made a remarkable recovery with the commencement of high-dose steroids slowly tapered over the course of weeks. Post-discharge outpatient visits showed back-to-baseline neurological and psychiatric status. It is important to note that both SREAT and NPSLE are rare diagnoses. They both overlap in many areas including their similar presentation, the lack of confirmatory tests, the diagnostic challenges, and their excellent response to steroids.

## Introduction

Hashimoto encephalopathy (HE), or rather the less misleading nomenclature, steroid-responsive encephalopathy associated with autoimmune thyroiditis (SREAT), is a rare syndrome associated with changes in mental status, confusion, seizures, and psychotic symptoms. SREAT diagnosis is challenging, given the nonspecificity of its symptoms and the cross-over in presentation with multiple diseases of metabolic, autoimmune, infectious, neurological, and psychiatric nature. In addition, the absence of confirmatory testing makes the diagnosis one of exclusion [1].

Neuropsychiatric systemic lupus erythematosus (NPSLE) on the other hand is a common but often under-diagnosed manifestation of SLE. There is a wide spectrum of both central nervous system (CNS) and peripheral nervous system (PNS) syndromes associated with NPSLE. The diagnosis is often a challenge as there are no confirmatory tests approved for the diagnosis. However, lupus flare markers including anti-double-stranded deoxyribonucleic acid antibody (anti-dsDNA Ab) titers, complement levels, leukocytes, and platelet levels along with magnetic resonance studies can help [2].

We present the case of a known SLE and Hashimoto thyroiditis patient who had presented with psychotic and encephalopathic symptoms. In the context of multiple known comorbidities pertinent to her clinical presentation, several pivotal diagnostic indices have been either conspicuously absent or inconclusive. Consequently, the multidisciplinary teams overseeing the patient's care have begun to entertain the possibility of an alternative diagnosis.

## Case presentation

A 27-year-old female patient was brought in by her family to the emergency department with a concern regarding her bizarre behavior and altered mental status that had been ongoing for a few days. Collateral history from a first-degree relative revealed that she had been very talkative and irrelevant in her speech, disinhibited, euphoric, exhibiting flight of ideas, and guarded and suspicious towards her siblings. She had also been energetic and reported difficulty falling asleep for five days prior to her presentation. Her psychiatric evaluation indicated inattentiveness with poor concentration and disorientation, in addition to paranoia and commanding auditory hallucinations. She was delusional and had thought-broadcasting. She had obsessions about cleanliness, along with occasional mood swings. About two to three years prior to that, she had a similar presentation which improved after symptomatic treatment with a low-dose antipsychotic, without receiving a specific diagnosis. 

Her past medical history included SLE, complicated with class V lupus nephritis. She was maintained on regular low-dose prednisolone (5 mg daily), mycophenolate mofetil (750 mg two times a day (BID)), and belimumab (200 mg subcutaneous (SC) weekly). She also had recurrent pulmonary embolisms and was on lifelong warfarin. There was no serological evidence of antiphospholipid syndrome with negative antiphospholipid antibodies. She had been diagnosed with hypothyroidism secondary to Hashimoto thyroiditis and was maintained on levothyroxine for many years. Her surgical history included bilateral hip replacement surgery due to bilateral femoral head avascular necrosis.

On presentation, the patient was afebrile, with a blood pressure of 135/83 mmHg, a heart rate of 99 beats/minute, a respiratory rate of 18 breaths/minute, and an oxygen saturation of 94% on ambient air. The patient was alert but only oriented to person with a Glasgow Coma Scale of 14/15. Physical examination did not reveal any rashes, oral ulcers, or alopecia. Eye examination was unremarkable. There was no joint tenderness or swelling. Auscultation of the heart did not reveal any murmurs or friction rub. Lung fields were resonant on percussion and clear on auscultation with no added sounds. There were no focal neurological signs.

Her initial labs are included in Table 1.

**Table 1:** Initial labs AST: aspartate aminotransferase; ALT: alanine transaminase; VBG: venous blood gas; TSH: thyroid-stimulating hormone; T4: tetraiodothyronine; dsDNA Ab: double-stranded deoxyribonucleic acid antibody; INR: international normalized ratio; pCO2: partial pressure of carbon dioxide; pO2: partial pressure of oxygen; HCO3: bicarbonate; IgM: immunoglobulin M; IgG: immunoglobulin G

Variable	Reference range	Result
Hemoglobin (g/dl)	11.6-14.8	9.3
White blood cell count (per μl)	4.5-11.0	5.3×10^9^/L
Neutrophils (%)	55-70	79.10
Lymphocytes (%)	20-40	12.10
Monocytes (%)	2.0-10.0	8
Eosinophil (%)	0.0-8.5	0.6
Platelet count (per μl)	140,000-400,000	534,000
AST (IU/L)	≤32	23
ALT (IU/L)	≤33	11
Albumin (g/L)	25-52	14
Sodium (mmol/L)	135-145	135
Potassium (mmol/L)	3.6-4.8	4.1
Chloride (mmol/L)	101-108	104
Calcium corrected (mmol/L)	2.23-2.58	2.58
Creatinine (micromol/L)	61-106	44
Urea nitrogen (mmol/L)	2.80-8.10	1.5
C‐reactive protein (mg/dl)	≤5	12.5
Procalcitonin (ng/ml)	≤0.50	0.05
pH VBG	7.35-7.45	7.36
pCO2 VBG (mmHg)	35.0-45.0	44
pO2 VBG (mmHg)	25.0-40.0	24.8
HCO3 VBG (mmol/L)	22-26	25
Lactic acid VBG (mmol/L)	0.5-2.2	0.5
TSH (milli IU/L)	0.270-4.200	82.7
Free T4 (pmol/L)	12.0-22.0	10.6
C3 complement (g/L)	0.9-1.8	1.38
C4 complement (g/L)	0.1-0.4	0.26
dsDNA Ab (IU/mL)	<26	127.1
Beta 2 glycoprotein IgM (CU)	<20	<1.1
Beta 2 glycoprotein IgG (CU)	<20	15.5
Cardiolipin IgM (CU)	<20	<1.0
Cardiolipin IgG (CU)	<20	<2.6
INR	0.7-1.1	2.6

Further investigations revealed a negative urine toxicology screen. Sepsis workup including blood cultures was negative. Inflammatory markers were unremarkable. Imaging showed a normal brain magnetic resonance imaging (MRI) and a negative magnetic resonance angiography (MRA)/magnetic resonance venography (MRV) study for cerebral arterial or venous pathology. Cerebrospinal fluid (CSF) analysis only showed a slight elevation in immunoglobulin G (IgG) level but was otherwise unremarkable.

Subsequent labs are included in Table 2.

**Table 2:** Subsequent labs THC: tetrahydrocannabinol; EDDP: 2-ethylidene-1,5-dimethyl-3,3-diphenylpyrrolidine; CSF: cerebrospinal fluid; IgG: immunoglobulin G; LDH: lactate dehydrogenase; RBC: red blood cell

Variable	Reference range	Result
C-reactive protein (mg/L)	<5.00	4.50
Procalcitonin (ng/ml)	<0.50	0.05
Urine ethanol level (mmol/L)	0.0-2.2 mmol/L	<2.2
Urine amphetamine screen	Undetected	Undetected
Urine barbiturate screen	Undetected	Undetected
Urine benzodiazepine screen	Undetected	Undetected
Urine cannabis screen (THC)	Undetected	Undetected
Urine cocaine screen	Undetected	Undetected
Urine methamphetamine screen	Undetected	Undetected
Urine opiate screen	Undetected	Undetected
Urine tricyclic screen	Undetected	Undetected
Urine meth metabolite screen (EDDP)	Undetected	Undetected
CSF appearance	Comment	Slightly bloody
CSF RBC (per liter)	0-5×10^6^	2,150×10^6^
CSF nucleated cells (per liter)	0-5×10^6^	4×10^6^
CSF IgG (mg/L)	10-30	48
CSF protein (g/L)	0.15-0.45	0.20
CSF glucose (mmol/L)	>2.1	3.0
CSF LDH (IU/L)	<25	19

NPSLE was high on the differential diagnosis, but the patient's lupus activity markers did not point towards an exacerbation. Her SLE Disease Activity Index (SLEDAI) score during this admission was found to be 12 mainly driven by these neurological symptoms. Although her anti-dsDNA levels were elevated at 127 IU/mL, that was much lower than her previous lupus flares, which were also associated with significantly low white blood cell (WBC) and low complement levels. A year prior to this presentation, the patient presented to the clinic with worsening proteinuria due to a lupus flare. At that time, her WBC was low at 1.95×10^9^/L and C3 was 0.52 g/L, while her C4 was 0.02 g/L. The patient had very high anti-dsDNA levels of >666.9 IU/mL (see Table 1 for normal reference ranges). Given the patient's insignificant lupus activity markers on this admission and the lack of significant abnormalities in brain MRI and CSF analysis, NPSLE was considered a less likely cause of her presentation.

The patient was found to be non-adherent to levothyroxine therapy, as inferred from familial history and corroborated by markedly elevated thyroid-stimulating hormone (TSH) levels (82.7 milli IU/L) alongside mildly diminished free tetraiodothyronine (T4) levels (10.6 pmol/L) upon hospital admission. Myxedema psychosis was another important differential. However, T4 levels were only mildly depressed, and she did not respond to levothyroxine therapy for 12 days making that possibility also unlikely.

An alternative diagnosis for her current presentation was sought. Anti-thyroid antibodies were positive including thyroid peroxidase (TPO) antibodies of 42 IU/L and anti-thyroglobulin antibodies (TgAB) at 533 IU/ml. The presence of SREAT is often described in the setting of euthyroid status along with anti-thyroid antibodies. A working diagnosis of SREAT was made, and the patient was then treated for SREAT with pulse steroid therapy (1 gram of intravenous methylprednisolone daily) for five days, followed by a slow tapering oral prednisolone regimen. The patient started showing improvement post-treatment as early as day 3, after finishing the five-day pulse steroid therapy. She continued to make a recovery and was discharged home just two weeks later, with a back-to-baseline mental state. Follow-up visits demonstrated continued resolution.

## Discussion

The prevalence of SREAT is estimated at 2/100,000 [3]. SREAT has a female predominance. The literature review reported that it acutely presents with neurological symptomatology, while a more chronic form would rather present with psychotic features. Neurological manifestations include encephalopathy, hyperreflexia, tremors, seizures non-responsive to anti-epileptic medications, stroke-like symptoms, coma, presenile dementia, ataxia, and isolated myelopathy [4]. Visual hallucinations are the most reported psychotic symptoms reported with up to 90% frequency [5]. SREAT is associated with elevated levels of anti-TPO antibodies in the serum, although no direct causation has been demonstrated. The diagnosis of SREAT is a diagnosis of exclusion. Making the diagnosis necessitates the presence of anti-thyroid antibodies. The response to steroids illustrated in HE led to the more acceptable nomenclature of SREAT [1].

The pathogenesis of SREAT is unclear; however, elevation in anti-TPO, anti-thyroglobulin, or anti-thyrotropin receptors was apparent in patients. Some studies believed that antibodies have a direct effect on the pathogenesis of the disease as the resolution of symptoms depicted a reduction in the antibody levels, but other researchers disagreed [4]. Antibody titers, however, do not correspond to the severity of presentation, but may begin to rise if treatment is insufficient. Antibody levels also do not affect the course or duration of the treatment [6]. With respect to the pre-existing thyroid disease, it was of great interest to note that patients with SREAT often had normal T3-T4 levels with normal or close to normal TSH levels. Our patient's TSH were elevated due to poor adherence to thyroid replacement medications; this was also observed in other case reports [7].

Diagnosing SREAT is often challenging as it is a diagnosis of exclusion which requires symptomatology of encephalopathy, along with elevated anti-thyroid antibodies. In 1966, Lord Brain and his colleagues were the first to report Hashimoto's disease and encephalopathy in a 49-year-old gentleman [1]. Peschen-Rosin et al. in 1999 were the first to set a diagnostic criterion for SREAT, which included myoclonus, generalized seizures, psychiatric manifestations, or localized neurological deficits in addition to an abnormal electroencephalogram (EEG), a rise in thyroid antibodies, and CSF analysis with an elevation in protein [8]. It was also proposed that an excellent response to corticosteroid treatment and normal brain MRI are strongly in favor of SREAT. More recent literature, however, suggests that in addition to the previously stated criteria, the presence of anti-thyroid antibodies in the serum or in the CSF is rather stronger than corticosteroid responsiveness as treatment response may only occur in half of the inflicted patients [9]. Abnormal thyroid function tests may also suggest the possibility of SREAT, although they can frequently be normal. Anti-TPO antibodies are present in 95% of patients, while anti-thyroglobulin is present in 60-80% of patients [10].

SREAT management is mainly centered on corticosteroid administration, treating the underlying thyroid pathology, and anti-epileptics if seizures are present [11]. Guidelines regarding corticosteroid dosing are yet to be established. Pulse steroids are typically given over five days followed by a regular dose, which is then tapered down gradually over months with close follow-up. Tapering slowly is usually beneficial in reducing relapses. Intravenous methylprednisolone 1 gram/day usually results in marked improvement over the course of one week. Other tried therapies include azathioprine and cyclophosphamide. Rituximab may also be considered as it was reported to show prolonged improvement. Intravenous immunoglobulin (IVIG) has been successful in both adults and pediatric patients. Plasma exchange, however, has not shown significant improvement, although it can reduce autoantibody levels [1].

Our case was quite challenging to diagnose, as our patient was a 27-year-old woman already diagnosed with SLE. Differential diagnosis included NPSLE, hypothyroidism-related encephalopathy (myxedema psychosis), and SREAT. NPSLE can present with neuropsychiatric manifestations including confusion, psychosis, seizures, cognitive dysfunction, and movement disorder, along with other nonspecific symptoms, all of which cross over with SREAT symptoms [12].

Neuropsychiatric involvement is a complex and often challenging clinical manifestation of lupus. Prevalence ranges from 14% to 80% in adults with SLE [2]. The American College of Rheumatology (ACR) has defined 19 neuropsychiatric syndromes (12 involving the CNS and seven involving the PNS). Acute confusional state and psychosis are among the 12 CNS syndromes and are the most relevant to our patient's presentation. An acute confusional state manifests as a fluctuating level of consciousness and disorientation. It varies and can range from subtle cognitive decline to the most pronounced acute confusional state [13]. Psychosis is a severe disturbance in the perception of reality characterized by delusions and/or hallucinations.

There are two main pathogenic mechanisms described for NPSLE. The first is the autoimmune-mediated neuroinflammatory pathway with complement activation, increased permeability of the blood-brain barrier, intrathecal migration of neuronal antibodies, and local production of immune complexes. This is usually associated with diffuse neuropsychiatric manifestations, for example, cognitive decline, acute confusional states, or mood disorders.

The other is the ischemic pathway, mediated by antiphospholipid antibodies, other immune complexes, and intravascular thrombosis. This tends to be associated with focal NPSLE syndromes such as cerebrovascular events and seizures [14-16]. Our patient's antiphospholipid antibodies profile was negative, and even if positive, it would have been more related to focal rather than diffuse NPSLE syndromes. Several antibodies are associated with NPSLE especially with the neuroinflammatory pathway including anti-neuronal, anti-NR2, anti-ribosomal P, and anti-endothelial antibodies [17].

NPSLE can present as a diagnostic challenge. It usually depends on the combination of clinical manifestations, abnormalities on CSF analysis, neuropsychological testing, neuroimaging, and exclusion of other causes that can cause similar presentations. To date, there are no specific serum or CSF biomarkers for the diagnosis of NPSLE. Although NPSLE can be the presenting feature in a minority of lupus patients, it usually happens in the presence of serologically and clinically active lupus. The lupus activity markers including complement C3 and C4 levels and anti-dsDNA Ab titers should therefore be checked in all suspected cases.

Diagnostic biomarkers in the serum and CSF along with neuroimaging are still an area under research. For patients with established SLE, antibodies such as antiphospholipid, anti-neuronal, anti-ribosomal P, and anti-NR2 can be helpful in the diagnosis of NPSLE. Elevation in interleukin-6 in the CSF also shows a positive correlation to diffuse NPSLE especially in acute confusional state. Neuroimaging studies including MRI, MRV, and MRA are the preferred modality for the diagnosis of NPSLE; however, half the patients may have a normal MRI [2].

Acute confusional state as part of NPSLE can have a similar clinical presentation to other causes of encephalitis. Low complement C3 and C4 levels, high anti-dsDNA Abs, and CSF analysis demonstrating low to normal glucose, pleocytosis and positive oligoclonal bands, and elevated IgG index can suggest the possibility of NPSLE [18]. Our patient had normal complement C3 and C4 levels and only mild elevation in anti-dsDNA antibodies. Our patient's CSF was not tested for the presence of anti-thyroid antibodies, as the diagnosis was not yet considered during the time of the lumbar puncture. Although our patient's anti-dsDNA was elevated, the elevation was much milder than her prior flare-ups, and other flare markers including a reduction in complement levels and leukopenia were absent. In the argument regarding the elevation in IgG, although it can be present in NPSLE, other case reports of SREAT have demonstrated a rise in IgG in the CSF of patients [19]. An elevation in CSF protein levels can serve as an indicator of SREAT; however, it's notable that approximately 30% of patients, including our own case, do not exhibit this characteristic [20].

Regarding neuroimaging in the diagnosis of SREAT, the literature has shown that MRI is unremarkable in approximately half of the patients. In the remaining patients, the most common findings included diffuse cerebral atrophy, an increase in signal on T2-weighted and fluid-attenuated inversion recovery (FLAIR) images in the white matter of the subcortical regions, and dural enhancement [19]. EEG findings may include nonspecific delta or theta waves. Epileptiform discharges, repetitive focal spikes or sharp waves, photo-myogenic response, photo-paroxysmal response, and generalized biphasic or triphasic waves were also reported. It is known that 60-70% of SEART cases are accompanied by seizures [21].

Newer autoantibodies in SREAT as proposed by Yoneda et al. are still under research; testing for autoantibodies like the amino (NH2)-terminal of α-enolase (NAE) in the serum may be useful in the diagnosis of SREAT [20].

We are aware of the limitations posed in this case. The patient received multiple treatments for a variety of issues, including antipsychotics by our psychiatry team, which had been increasing in dosing due to poor response, thyroid hormone replacement for her hypothyroidism, and the high-dose steroids, which would have alleviated NPSLE if it had been the underlying cause. However, the prompt response to the steroids as compared to all the other treatments provided, and the aforementioned rationale indicating a less likely probability for the diagnosis of NPSLE, led us to believe that SREAT was the most likely cause of her symptoms.

## Conclusions

The challenge of considering SREAT instead of NPSLE in a patient with a confirmed diagnosis of SLE highlights the critical need for multidisciplinary collaboration, thorough utilization of laboratory and imaging assessments, and maintaining a flexible approach to differential diagnosis, especially in cases with atypical presentations.

Given SREAT's excellent response to treatment, it is important to consider it in the differential diagnosis of altered mental status even when other potential explanations exist. It would be interesting to study the presence of thyroid antibodies in SLE, specifically in NPSLE patients, and to determine if SREAT explains some of the NPSLE cases. Further research in that area can be of great value.
